# Supplementary material for: Drug repositioning by merging active subnetworks validated in cancer and COVID-19
Source: Sci Rep. 2021 Oct 6;11:19839. doi: 10.1038/s41598-021-99399-2 (PMC8494853; doi:10.1038/s41598-021-99399-2)
Supplement: Supplementary file 1 — Supplementary Information. [file 41598_2021_99399_MOESM1_ESM.pdf]

Drug Repositioning by Merging Active Subnetworks Validated in Cancer and COVID-19 - Supplementary Material -

Marta Lucchetta[1,2] and Marco Pellegrini[1,*]

[1]Institute of Informatics and Telematics (IIT), CNR, Pisa, 56124, Italy
[2]Department of Biotechnology, Chemistry and Pharmacy, University of Siena, Siena, 53100, Italy
[*]marco.pellegrini@iit.cnr.it

Contents

1 Drug perturbation databases **1**

2 Drug name and aliases **2**

3 CMAP algorithm **2**

4 DrugMerge results on Asthma **2**

5 DrugMerge results on Rheumatoid Arthritis (RA) **3**

6 Related work **5**

7 Supplementary Tables **8**

1 Drug perturbation databases

To predict the potentially repurposable drugs in each active subnetwork (one for each disease and each method), we used four drug perturbation databases available in Enrichr (`https://maayanlab.cloud/Enrichr/#stats`). These databases are called 'Drug_Perturbations_from_GEO_2014', 'DrugMatrix',
'LINCS_L1000_Chem_Pert_down', 'LINCS_L1000_Chem_Pert_up', 'Old _CMAP_down', and 'Old_CMAP_up'. The GEO- and DrugMatrix-related analysis has been performed in June 2020, that one for L1000 and CMAP has been performed in January 2021. Each database includes several drugs and the corresponding genes perturbed by the drug, distinguishing if these genes are up- or down-regulated. The Drug Perturbations database from GEO was compiled using experiments from GEO [1] where gene expression levels were measured before and after the administration of a drug. This library has drug signatures for several tissues, and species including the *homo sapiens*, *mus musculus* and *rattus norvegicus*. In total, there are 701 terms and 132 unique drugs (where we do not consider the species, the tissues, and the modularity of the genes). DrugMatrix (DM) [2], [3] is one of the largest rat toxicogenomics databases and has drug signatures available on liver, heart, kidney,

thigh muscle, and primary hepatocytes. In total, it includes 7876 terms and 656 unique drugs. The Connectivity Map (also called CMAP) [4], [5] is a collection of gene expression data from five different human cells perturbed with many chemicals and genetic reagents. In total, CMAP applied 1309 compounds yielding 6100 profiles. The Connectivity Map project entered a most recent version of CMAP, as part of NIH's Library of Integrated Network-Based Cellular Signatures (LINCS) program, called LINCS-L1000. It comprises $\sim$ 5000 genetic perturbations and $\sim$ 15000 perturbations induced by chemical compounds [6] across 98 different cell lines. The two CMAP projects differ also in sequencing platforms. The LINCS-L1000 project replaced the Affymetrix GeneChips used by the original CMAP with Luminex bead arrays [7], which has been developed to facilitate rapid, flexible, and high-throughput gene expression profiling at lower costs. Using the *enrichR* package, in the L1000 database, we obtained 33132 terms and 4117 compounds.

2 Drug name and aliases

We handle drug aliases by using the MESH[1] database. Moreover, we identified commercial drug names with their principal active ingredient. Ambiguous cases were checked by hand against the pharmacological literature and DrugBank records (https://go.drugbank.com/).

3 CMAP algorithm

To compare the DrugMerge performance in the four benchmark diseases (asthma, rheumatoid arthritis, prostate cancer, and colorectal cancer), we used the *PharmacoGx* [8] R package, which performs the Connectivity Map (CMAP) analysis [4]. For each disease, we used the differentially expressed genes (DEGs) as gene input to identify drugs with therapeutic potential in each disease. In particular, we used the *downloadPSet* function to download the CMAP database, and the *drugPerturbationSig* function to identify differential gene expressions induced by drug treatment. Finally, the *connectivityScore* function compares drug signatures against disease signatures (DEGs), by assigning connectivity score and p-value for each drug. The connectivity score determines the correlation between the drug and disease signatures and it ranges from -1 to 1. Since we are looking for disease treatments or drug repurposing, the CMAP drugs should be anti-correlated with disease signatures. For this reason, we ordered the final CMAP drugs according to the increasing order of the connectivity score (from negative to positive values). We also filtered the CMAP drugs according to p-value < 0.05 and we used both lists with or without p-value filtering. Starting from these lists, we calculated the RHR and precision@20 with z-score and p-values associated. After that, we selected those results with the best values of RHR and compare them with values detected by DrugMerge as reported in Figure 4 of the main text and Table S6.

4 DrugMerge results on Asthma

Figure 1 and Supplementary Table S1 show that DrugMerge on the GEO Drug dataset finds one clinically relevant drug (prednisolone) in first position both when all drugs are used and when only FDA approved drugs are used. The precision@20 is not statistically significant, but the RHR of this single hit is very significant. A closer look at the GEO data and at the TTD records reveals that prednisolone is the only drug from TTD present in the GEO dataset. The initial list of differentially expressed genes is measured from a cohort of patients with acute asthma with respect to healthy patients, and interestingly the hit drug prednisolone is used specifically for acute asthma[2]. The DrugMerge algorithm does not find any hit in the L1000 drug dataset. An examination of the L1000

[1] https://www.nlm.nih.gov/mesh/
[2] https://www.mayoclinic.org/diseases-conditions/asthma/in-depth/asthma-medications/art-20045557

data shows that only 5 asthma-related drugs as reported by TTD are in the L1000 data (budesonide, fluticasone propionate, mometasone furoate, beclomethasone, and colforsin) which are used to treat chronic asthma or mild/moderate persistent asthma. Similar considerations hold for the TTD drugs present in CMAP Drug data. Thus DrugMerge appears to be able to differentiate the acute vs chronic forms of the asthma disease and rank higher drugs that are fit for the subclass of asthma patients assessed in the data set (acute asthma patients).

Among the top positions in the ranking reported in Supplementary File S7, we find drugs with known effects in asthma patients or animal models of asthma, and one of these went into clinical trial stage: deferasirox [9], rosiglitazone [10], valproic acid[3], dexamethasone [11], celecoxib [12], and tamoxifen [13].
Rosiglitazone has been tested on a murine model of chronic asthma [10], suggesting that its intranasal administration can prevent air way inflammation.
A comparison study between dexamethasone versus hydrocortisone in severe acute pediatric asthma [11] showed that the mean length of hospitalization in children receiving dexamethasone was significantly shorter than those receiving hydrocortisone. Celecoxib is a COX-2 inhibitor and nonsteroidal anti-inflammatory drug.
A study of 33 asthma patients [12] demonstrated that celecoxib is a suitable drug in aspirin-induced and/or nonsteroidal anti-inflammatory drug-induced asthma patients.
Tamoxifen is an estrogen receptor modulator mainly used for the treatment of breast cancer, but it has also been reported to have anti-inflammatory activity. A recent study [13] has tested tamoxifen on three different mouse models, showing that tamoxifen can reduce inflammatory infiltration of neutrophils in the airways.

For the remaining drugs in the top positions, we focus on their mechanism of action and we search in literature if this can have relevance in asthma. Using this criterion, we find captopril, letrozole, decitabine, imatinib, and probucol as relevant drugs.
Captopril belongs to the class of drugs known as angiotensin-converting enzyme (ACE) inhibitors and is used primarily to lower high blood pressure (hypertension). It has been proved that asthmatic subjects with comorbid hypertension display evidence of enhanced asthma morbidity [14].
Letrozole inhibits aromatase, an enzyme that catalyzes the synthesis of estrogen. Asthma prevalence and severity are greater in women than in men, suggesting this is in part related to female steroid sex hormones, such as estrogen. Estrogen receptors are found on numerous immunoregulatory cells and estrogen's actions skew immune responses toward allergy [15].
Decitabine is a hypomethylating agent and is used to treat myelodysplastic syndromes and acute myeloid leukemia. Yang et al. [16] demonstrated that DNA methylation in specific gene loci are associated with asthma and suggest that epigenetic changes might play a role in establishing the immune phenotype associated with childhood asthma.
Imatinib is a tyrosine kinase inhibitor. Anti-inflammatory effects of tyrosine kinase inhibitors have been reported in animal models of allergic asthma, suggesting that this kind of drug can be a very attractive strategy for the treatment of asthma [17].
Finally, probucol lowers the level of cholesterol through the inhibition of cholesterol synthesis and deletion of cholesterol absorption. Ramaraju et al. [18] found a modest but significant association between higher levels of serum cholesterol and asthma.

5 DrugMerge results on Rheumatoid Arthritis (RA)

Figure 2 and Supplementary Table S2 show that DrugMerge finds significant rankings in several drug databases (L1000, DrugMatrix, GEO), with GEO attaining the best p-value, using several algorithms, both when all drugs are considered or when only FDA-approved drugs are considered. For the GEO dataset Supplementary Table S7, we find in first position the clinically relevant drug

[3]In clinical trial https://clinicaltrials.gov/ct2/show/NCT00153270

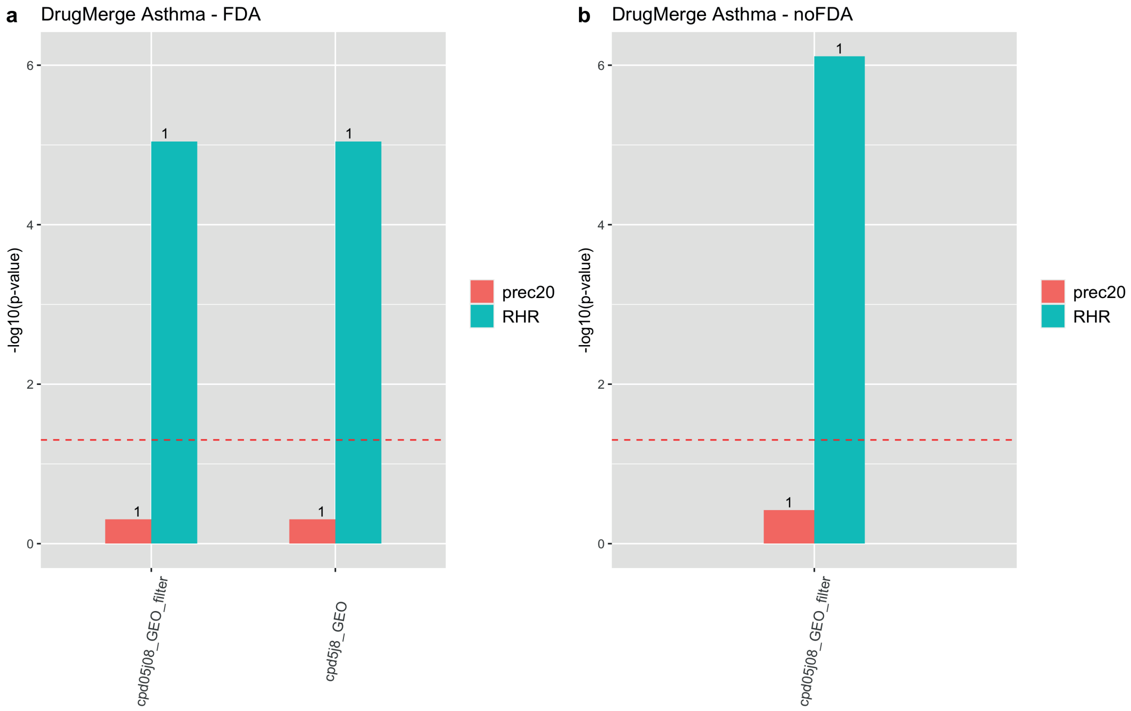

Figure S 1: DrugMerge performance on Asthma data. The bars represent the $-log_{10}(pvalue)$ with respect to the precision@20 (red), and the RHR (light blu). On the x-axis, different algorithms or combinations of them are shown. The numbers on the top of the bars show the absolute values of precision@20 or RHR. The dotted red line represents the limit of significance ($-log_{10}(0.05)$). All the bars above the dotted line show a significant p-value. The a) panel represents the DrugMerge performance when only FDA-approved drugs are considered; b) when all drugs (without any FDA filtering) are considered. The plot has been generated by the *ggplot2* [19] R package.

dexamethasone. Other drugs in use according to the TTD records that appear in the top twenty positions are: imatinib, methylprednisolone, and prednisolone.

Among the top positions in the ranking, we find drugs with known effects in rheumatoid arthritis patients or animal models of rheumatoid arthritis, and one of these went into clinical trial stage: rosiglitazone[4], pioglitazone [20], estradiol [21], captopril [22], vitamin c [23], baclofen [24], deferasirox [25], decitabine [26], sirolimus [27], hydrocortisone[28], paclitaxel [29], and ethinylestradiol [30].
Suke et al. [20] studied the effects of combined pioglitazone and prednisolone on adjuvant-induced arthritis in rats. This study suggested that the combination of these two drugs was effective in modulating the inflammatory response and suppress arthritis progression.
Estradiol is an estrogen steroid female hormone, and estrogens have a direct action upon the immune system. The role of the estrogens in rheumatoid arthritis has been studied here [21].
Captopril is prescribed for hypertension but it has immunosuppressant activity, as well. Therefore, captopril was considered a potential slow-acting drug for treating rheumatoid arthritis as demonstrated in [22].
Vitamin C is a vitamin found in various foods and it is important for immune system function. It has also been studied the role of the vitamin c in treating pain [31], in particular, an administration of high-dose vitamin C in patients with rheumatoid arthritis showed a complete decrease in pain

[4] In clinical trial https://clinicaltrials.gov/ct2/show/NCT00379600

[23].
Baclofen is a medication used to treat muscle spasticity. Huang et al. [24] investigated the effects of baclofen in murine collagen-induced arthritis, proving that baclofen alleviated the clinical development of arthritis.
Decitabine has already been found in asthma analysis and as mentioned before, it inhibits DNA methylation. Petralia et al. [26] have studied the effect of decitabine in a murine model of rheumatoid arthritis and have demonstrated that decitabine administration was associated with a significant improvement of the clinical condition.
Sirolimus, also known as rapamycin, has immunosuppressant functions and is used to prevent rejection in organ transplants. Wen et al. [27] studied the safety, tolerance, and efficacy of sirolimus in patients with active RA treated with low-dose sirolimus combined with original therapy. They showed that this therapy alleviates clinical symptoms and decreases the immunosuppressive applications in patients with active RA.
Hydrocortisone is a treatment for acute episodes of rheumatic disorders, including rheumatoid arthritis [5].
Paclitaxel is an anticancer agent and is classified as a microtubule-stabilizing agent. Kurose et al. [29] studied the effects of paclitaxel on cultured synovial cells from patients with rheumatoid arthritis. The data suggest paclitaxel as a possible therapy for RA.
Ethinylestradiol is an active estrogen and component of birth control pills. Subramaniam et al. [30] studied the effectiveness of ethinylestradiol in treating collagen-induced arthritis mice, noticing a decreased proliferation and secretion of pro-inflammatory factors.

Focusing on the action mechanism of the remaining drugs, we find some links with rheumatoid arthritis, such as amoxicillin [32], and niacin [33].
Amoxicillin is an antibiotic used to treat several bacterial infections. Since the 1930s, RA has been treated with antibiotics and there have been several reports in the literature indicating that periodontal pathogens are a possible cause of the disease [32].
Niacin, more commonly known as vitamin B3, is a precursor of the coenzymes nicotinamide-adenine dinucleotide (NAD+). Recent studies have identified potential therapeutic approaches for boosting NAD+ to treat rheumatologic diseases, including rheumatoid arthritis. In particular, they focused on the enzymatic activity of CD38, one of the main enzymes in NAD+ catabolism [33].

6 Related work

Computational drug repositioning is a burgeoning field of research (see. e.g. [34], [35] [36], [37], [38], [39], [40], [41], [42], [43]) receiving much attention for its high potential impact on coping with present and future pandemic events by novel pathogens [44] [45].

For the four benchmark diseases considered in our study, studies on repurposed drugs for tumors are reported in [46], [47], [48], for asthma in [49], and for rheumatoid arthritis in [50], and [51].

A survey focusing on repurposing anti-cancer drugs for covid19 is in [52]. Computational drug repositioning is gaining much attention during the current covid19 pandemic as reported in [53].

Network-based drug repositioning [54] is an approach that leverages on building and analyzing various types of biological networks integrating several layers of 'omic' data [55]. Also, drug perturbation data bases play a central role in computational drug profiling [56], [4], [57], [3]. Next, we focus on some results in the lines of research that are most relevant for our study.

Taguchi et al. [58] apply an unsupervised method based on tensor decomposition to perform feature extraction of gene expression profiles in multiple lung cancer cell lines infected with severe acute respiratory syndrome coronavirus 2 [59]. They thus identified, using Enrichr [60], drug candidates that significantly altered the expression of the 163 genes selected in the previous phase. Drug perturbation data is collected from GEO, DrugMatrix, L1000, and other repositories.

[5] https://www.drugs.com/monograph/hydrocortisone-systemic.html#ra

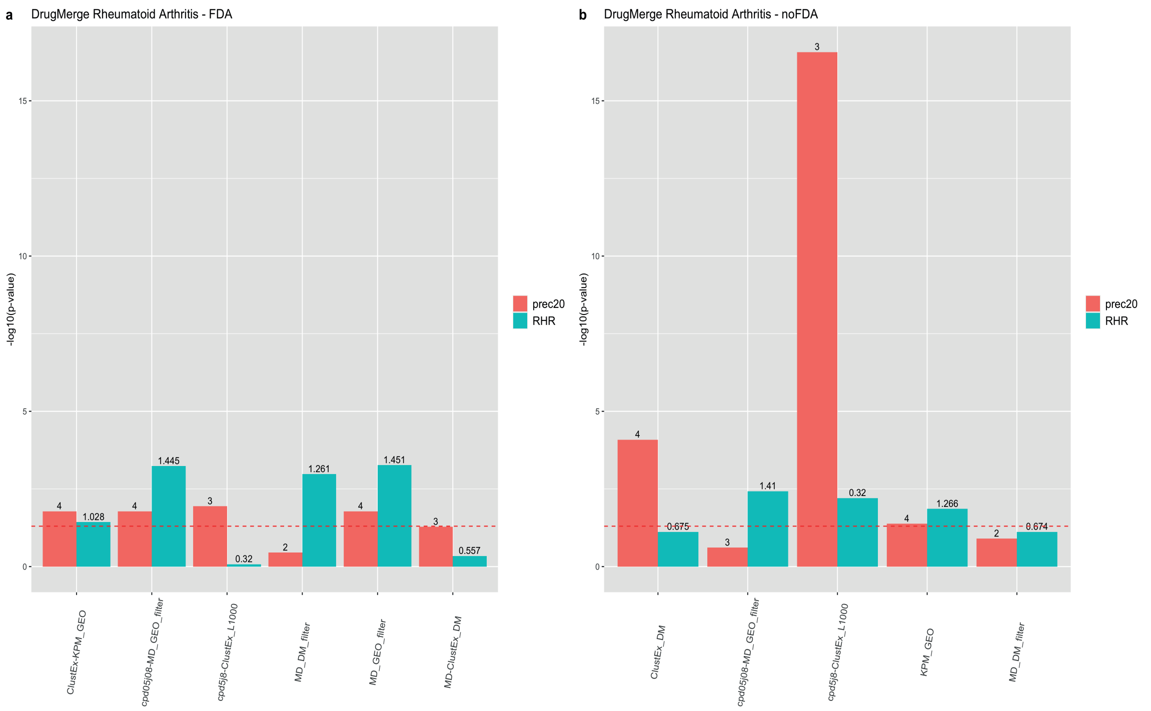

Figure S 2: DrugMerge performance on Rheumatoid Arthritis data. The bars represent the $-log_{10}(pvalue)$ with respect to the precision@20 (red), and the RHR (light blu). On the x-axis, different algorithms or combinations of them are shown. The numbers on the top of the bars show the absolute values of precision@20 or RHR. The dotted red line represents the limit of significance ($-log_{10}(0.05)$). All the bars above the dotted line show a significant p-value. The a) panel represents the DrugMerge performance when only FDA-approved drugs are considered; b) when all drugs (without any FDA filtering) are considered. The plot has been generated by the *ggplot2* [19] R package.

Ruiz et al. [61] build a multi-layer disease-gene-drug-pathway network and develop a random-walk-based score that captures indirect effects of drugs on diseases via commonly affected pathways. They validate their approach in a leave-one-out validation on a golden standard of 6000 drug-disease pairs commonly used in clinical practice. The paper, however, does not report on the specific application of this approach to COVID-19.

Fiscon et al. [62], [63] use an approach based on computing distances between regions of a large human interactome network affected by a disease (disease targets) and those affected by drugs (drug perturbations). This work relies on disease target similarities between a group of diseases including covid19, SARS-coV, MERS, and others. The final assessment of drug candidates for COVID19 is done with the C-map database.

Gysi et al. [64] define several proximity-based distance functions between covid19 human target proteins (as listed in [65]) and drug protein targets as listed in DrugBank [66], in order to prioritize repurposable drugs. Several refinement taking into account tissue specificity, drug action on gene expression levels (using data from [59]), comorbidities, and drug toxicity lead to a final list of 81 repurposable drugs for covid19. The main measure of performance chosen is the AUC of the predicted list versus the list of drugs currently employed in clinical trials for the treatment of COVID-19 as listed in https://www.clinicaltrials.gov/.

Our approach to performance evaluation is similar to the one in [64] since we also use drugs

in clinical trials as the golden standard for covid19. Thus we are both measuring how well our automated ranking systems come close to the collective wisdom of human experts that shortlisted existing drugs for repurposing on COVID-19 during 2020 based on a large variety of considerations such as available clinical and preclinical data, pharmacological background, guesses mechanism of action. However, we decided to use precision@20 and the Reciprocal Hit Ranking as quantitative measures since they are quite intuitive, more suitable for a ranking problem, and they handle more uniformly lists of candidate drugs that can span from a few dozen drugs to a few thousand.

Zhou et al. [67] also use proximity-based measures in biological networks to find a list of 16 repurposable drugs. It should be noticed that Zhou et al. use data collected from the family of human coronaviruses (HCoVs) to build the network, thus relying heavily on evolutionary conservation of relevant coding parts of the viral genome across the species in this family and on phylogenetic considerations.

Sadegh et al. [68] use the algorithm KeyPathwayMiner [69] to define the active subnetwork in an integrated COVID-19 resource network from CoronaVirus Explorer CoVex resource (https://exbio.wzw.tum.de/covex/). The main contribution of [69] is a resource (CoVex) that can be used interactively in many different scenarios and modalities to explore disease-gene-drug relationships for COVID-19.

Mall et al. [70] use deep learning induced vector embedding of drugs and viral proteins to predict drug-viral protein activity and propose a short list of 15 drugs potentially useful against COVID-19.

A second very popular approach uses knowledge of the 3-dimensional molecular configuration of proteins and drugs to simulate, via docking or molecular dynamics, the most promising drug-target bindings. Most research in docking-based drug repositioning starts with an assessment of one (or a few) protein acting as potential drug targets [71].

Seo et al. [72] take three dimensional structure of the main protease (Mpro) of SARS-CoV-2 as a target, and use docking simulations on a supercomputer to evaluate binding affinity between Mpro and drug candidates listed in the SWEETLEAD library and the ChEMBL database (19,168 molecules), thus shortlisting 43 drugs, which, after molecular dynamics simulations, are then reduced to 8. A similar docking-based screening is also used in [73], [74], [75], [76], [77], and [78].

Besides network-based and docking-based approaches there is a variety of other principles and approaches that have been used both *in silico* and *in vitro*: Luminescent cell viability assay and cell imaging [79] [80], morphological cell profiling [81], co-morbidity [82], deep learning [83], network proximity based [84], quantum mechanical scoring [85], multi-objective optimization [86], bipartite network projections [55], matrix factorization [87], machine learning [88], network node scoring [89] [90], semi-supervised learning [91], variants of gene set enrichment analysis (GSEA) [92], multi-omic integration [93].

A more direct experimental approach has been described in [65] and [94]. Gordon et al. [65] performed a direct proteomic assay to uncover 332 human proteins potentially interacting with 26 SARS-Cov2 proteins. Sixty-nine drugs targeting these proteins are then shortlisted as repurposable drugs for covid19, using a mix of chemo-informatic searches and expert advice. Note that in this setting a priority ranking is not provided.

A main theme along with drug ranking is that of drug combinations [95]. Often drug combinations offer lower toxicity and more effective disease treatment. Most drug combinations currently under consideration result from ad-hoc considerations, as screening drug combinations automatically incurs easily in a combinatorial explosion of cases to be considered. A few network-based principled approaches have been proposed and may be integrated into DrugMerge [96], [67], [97], [98], [99], [100], and [101].

While the final hallmark of success is the identification of a repurposed drug that can pass successfully all stages of drug approval for clinical use (see e.g. [102]), it is important to be able to perform *in silico* validation on intermediate results. A recent study by Brown and Patel [103] gives a critical assessment of the options for *in silico* drug validations, assessing the weak and strong points of each strategy.

7 Supplementary Tables

Supplementary Table S1. DrugMerge results on Asthma. The table reports the ranking function, the filter, drug perturbation data set, algorithmic configuration attaining best performance on the asthma DEG dataset. It is reported the reverse hits ranking and precision at 20, with corresponding z-scores and p-values.

Supplementary Table S2. DrugMerge results on Rheumatoid arthritis. Table similar to S1 for Rheumatoid Arthritis DEG dataset.

Supplementary Table S3. DrugMerge results on Colorectal cancer. Table similar to S1 for Colorectal Cancer DEG dataset.

Supplementary Table S4. DrugMerge results on Prostate cancer. Table similar to S1 for Prostate Cancer DEG dataset.

Supplementary Table S5. DrugMerge results on COVID-19. Table similar to S1 for COVID-19 DEG datasets.

Supplementary Table S6. CMAP algorithm vs DrugMerge. Table reporting comparative performance of CMAP and DrugMerge on four benchmark data sets (Asthma, Rheumatoid Arthritis, Prostate cancer, and Colorectal cancer). It is reported the reverse hits ranking and precision at 20, with corresponding z-scores and p-values.

Supplementary Table S7. Final drug lists. Drug rankings with best rhr pvalue performance for DrugMerge on four benchmark data sets (Asthma, Rheumatoid Arthritis, Prostate cancer, and Colorectal cancer), and COVID-19 (both with FDA approved only drugs, or all drugs).

Supplementary Table S8. Summary table of comparative results about COVID-19. Table reporting comparative performance of several published drug rankings on COVID-19. It is reported the reverse hits ranking and precision at 20, with corresponding z-scores and p-values.

Supplementary Table S9. Table of algorithms and aliases. Table mapping synthetic algorithms identifiers in Figures 5 and 6 with algorithmic configurations in Table S5.

References

[1] Tanya Barrett, Stephen E Wilhite, Pierre Ledoux, Carlos Evangelista, Irene F Kim, Maxim Tomashevsky, Kimberly A Marshall, Katherine H Phillippy, Patti M Sherman, Michelle Holko, et al. Ncbi geo: archive for functional genomics data sets—update. *Nucleic acids research*, 41(D1):D991–D995, 2012.

[2] Daniel L Svoboda, Trey Saddler, and Scott S Auerbach. An overview of national toxicology program's toxicogenomic applications: Drugmatrix and toxfx. *Advances in Computational Toxicology*, pages 141–157, 2019.

[3] Brigitte Ganter, Stuart Tugendreich, Cecelia I Pearson, Eser Ayanoglu, Susanne Baumhueter, Keith A Bostian, Lindsay Brady, Leslie J Browne, John T Calvin, Gwo-Jen Day, et al. Development of a large-scale chemogenomics database to improve drug candidate selection and to understand mechanisms of chemical toxicity and action. *Journal of biotechnology*, 119(3):219–244, 2005.

[4] Justin Lamb, Emily D Crawford, David Peck, Joshua W Modell, Irene C Blat, Matthew J Wrobel, Jim Lerner, Jean-Philippe Brunet, Aravind Subramanian, Kenneth N Ross, et al. The connectivity map: using gene-expression signatures to connect small molecules, genes, and disease. *science*, 313(5795):1929–1935, 2006.

[5] Justin Lamb. The connectivity map: a new tool for biomedical research. *Nature reviews cancer*, 7(1):54–60, 2007.

[6] Aliyu Musa, Laleh Soltan Ghoraie, Shu-Dong Zhang, Galina Glazko, Olli Yli-Harja, Matthias Dehmer, Benjamin Haibe-Kains, and Frank Emmert-Streib. A review of connectivity map and computational approaches in pharmacogenomics. *Briefings in bioinformatics*, 19(3):506–523, 2018.

[7] Nathaniel Lim and Paul Pavlidis. Evaluation of connectivity map shows limited reproducibility in drug repositioning. *BioRxiv*, page 845693, 2019.

[8] Petr Smirnov, Zhaleh Safikhani, Nehme El-Hachem, Dong Wang, Adrian She, Catharina Olsen, Mark Freeman, Heather Selby, Deena MA Gendoo, Patrick Grossmann, et al. Pharmacogx: an r package for analysis of large pharmacogenomic datasets. *Bioinformatics*, 32(8):1244–1246, 2016.

[9] Mongkhon Sompornrattanaphan, Thanachit Krikeerati, Chamard Wongsa, Torpong Thongngarm, and Kittika Yampayon. Successful deferasirox rechallenge and treating through reaction in a patient with challenge-proven mild immediate reaction: A case report. *Journal of Asthma and Allergy*, 13:557, 2020.

[10] Hwa Young Lee, Chin Kook Rhee, Ji Young Kang, Chan Kwon Park, Sook Young Lee, Soon Suk Kwon, Young Kyoon Kim, and Hyoung Kyu Yoon. Effect of intranasal rosiglitazone on airway inflammation and remodeling in a murine model of chronic asthma. *The Korean journal of internal medicine*, 31(1):89, 2016.

[11] Sedigheh Ebrahimi Bahador Sarkari. Comparative efficacy of dexamethasone versus hydrocortisone in severe acute pediatric asthma. *Iranian Journal of Allergy, Asthma and Immunology*, pages 159–160, 2007.

[12] C Martin-Garcia, M Hinojosa, P Berges, E Camacho, R Garcia-Rodriguez, and T Alfaya. Celecoxib, a highly selective cox-2 inhibitor, is safe in aspirin-induced asthma patients. *Journal of investigational allergology & clinical immunology*, 13(1):20–25, 2003.

[13] Agustín Mansilla, Jaime Soto, Claudio Henriquez, Amber R Philp, Marcus A Mall, Jose Sarmiento, and Carlos A Flores. Tamoxifen reduces inflammatory infiltration of neutrophils in the airways. *bioRxiv*, 2020.

[14] Sandra C Christiansen, Michael Schatz, Su-Jau Yang, Eunis Ngor, Wansu Chen, and Bruce L Zuraw. Hypertension and asthma: a comorbid relationship. *The Journal of Allergy and Clinical Immunology: In Practice*, 4(1):76–81, 2016.

[15] Rana S Bonds and Terumi Midoro-Horiuti. Estrogen effects in allergy and asthma. *Current opinion in allergy and clinical immunology*, 13(1):92, 2013.

[16] Ivana V Yang, Brent S Pedersen, Andrew Liu, George T O'Connor, Stephen J Teach, Meyer Kattan, Rana Tawil Misiak, Rebecca Gruchalla, Suzanne F Steinbach, Stanley J Szefler, et al. Dna methylation and childhood asthma in the inner city. *Journal of Allergy and Clinical Immunology*, 136(1):69–80, 2015.

[17] WS Fred Wong and Khai Pang Leong. Tyrosine kinase inhibitors: a new approach for asthma. *Biochimica et Biophysica Acta (BBA)-Proteins and Proteomics*, 1697(1-2):53–69, 2004.

[18] Karthikeyan Ramaraju, Srikanth Krishnamurthy, Smrithi Maamidi, Anupama Murthy Kaza, and Nithilavalli Balasubramaniam. Is serum cholesterol a risk factor for asthma? *Lung India: Official Organ of Indian Chest Society*, 30(4):295, 2013.

[19] Hadley Wickham, Winston Chang, and Maintainer Hadley Wickham. Package 'ggplot2'. *Create Elegant Data Visualisations Using the Grammar of Graphics. Version*, 2(1):1–189, 2016.

[20] Sanvidhan G Suke, Harsh Negi, PK Mediratta, BD Banerjee, and KK Sharma. Anti-arthritic and anti-inflammatory activity of combined pioglitazone and prednisolone on adjuvant-induced arthritis. *European journal of pharmacology*, 718(1-3):57–62, 2013.

[21] Maria Fernanda Romo-García, Martín Zapata-Zuñiga, José Antonio Enciso-Moreno, and Julio Enrique Castañeda-Delgado. The role of estrogens in rheumatoid arthritis physiopathology. In *Rheumatoid Arthritis-Other Perspectives towards a Better Practice*. IntechOpen, 2020.

[22] MFR Martin, F McKenna, HA Bird, KE Surrall, JS Dixon, and V Wright. Captopril: a new treatment for rheumatoid arthritis? *The Lancet*, 323(8390):1325–1328, 1984.

[23] AC Carr, MCM Vissers, and J Cook. Parenteral vitamin c relieves chronic fatigue and pain in a patient presenting with rheumatoid arthritis and mononeuritis multiplex secondary to cns vasculitis. *Case Rep Clin Path*, 2(2):57–61, 2015.

[24] Shichao Huang, Jianxin Mao, Bin Wei, and Gang Pei. The anti-spasticity drug baclofen alleviates collagen-induced arthritis and regulates dendritic cells. *Journal of cellular physiology*, 230(7):1438–1447, 2015.

[25] Rex J Polson, Ali Sm Jawad, Adrian Bomford, Hedley Berry, and Roger Williams. Treatment of rheumatoid arthritis with desferrioxamine. *QJM: An International Journal of Medicine*, 61(3):1153–1158, 1986.

[26] Maria Cristina Petralia, Emanuela Mazzon, Maria Sofia Basile, Marco Cutuli, Roberto Di Marco, Fabiola Scandurra, Andrea Saraceno, Paolo Fagone, Ferdinando Nicoletti, and Katia Mangano. Effects of treatment with the hypomethylating agent 5-aza-2́-deoxycytidine in murine type ii collagen-induced arthritis. *Pharmaceuticals*, 12(4):174, 2019.

[27] Hong-Yan Wen, Jia Wang, Sheng-Xiao Zhang, Jing Luo, Xiang-Cong Zhao, Chen Zhang, Cai-Hong Wang, Fang-Yuan Hu, Xiao-Juan Zheng, Ting Cheng, et al. Low-dose sirolimus immunoregulation therapy in patients with active rheumatoid arthritis: A 24-week follow-up of the randomized, open-label, parallel-controlled trial. *Journal of immunology research*, 2019, 2019.

[28] Edward W Boland. Rheumatoid arthritis—experiences with hydrocortisone (free alcohol) and hydrocortisone acetate. *California medicine*, 77(1):1, 1952.

[29] Akira Kurose, Wataru Yoshida, Masaaki Yoshida, and Takashi Sawai. Effects of paclitaxel on cultured synovial cells from patients with rheumatoid arthritis. *Cytometry: The Journal of the International Society for Analytical Cytology*, 44(4):349–354, 2001.

[30] Sandhya Subramanian, Micah Tovey, Michael Afentoulis, Aric Krogstad, Arthur A Vandenbark, and Halina Offner. Ethinyl estradiol treats collagen-induced arthritis in dba/1lacj mice by inhibiting the production of tnf-α and il-1β. *Clinical Immunology*, 115(2):162–172, 2005.

[31] Anitra C Carr and Cate McCall. The role of vitamin c in the treatment of pain: new insights. *Journal of translational medicine*, 15(1):1–14, 2017.

[32] Mesut Ogrendik. Antibiotics for the treatment of rheumatoid arthritis. *International journal of general medicine*, 7:43, 2014.

[33] Thais Ribeiro Peclat, Bo Shi, John Varga, and Eduardo Nunes Chini. The nadase enzyme cd38: an emerging pharmacological target for systemic sclerosis, systemic lupus erythematosus and rheumatoid arthritis. *Current opinion in rheumatology*, 32(6):488–496, 2020.

[34] Jiao Li, Si Zheng, Bin Chen, Atul J Butte, S Joshua Swamidass, and Zhiyong Lu. A survey of current trends in computational drug repositioning. *Briefings in bioinformatics*, 17(1):2–12, 2016.

[35] Jeremy M Levin, Tudor I Oprea, Sagie Davidovich, Thomas Clozel, John P Overington, Quentin Vanhaelen, Charles R Cantor, Evelyne Bischof, and Alex Zhavoronkov. Artificial intelligence, drug repurposing and peer review. *Nature Biotechnology*, 38(10):1127–1131, 2020.

[36] Gihanna Galindez, Julian Matschinske, Tim Daniel Rose, Sepideh Sadegh, Marisol Salgado-Albarrán, Julian Späth, Jan Baumbach, and Josch Konstantin Pauling. Lessons from the covid-19 pandemic for advancing computational drug repurposing strategies. *Nature Computational Science*, 1(1):33–41, 2021.

[37] Serena Dotolo, Anna Marabotti, Angelo Facchiano, and Roberto Tagliaferri. A review on drug repurposing applicable to covid-19. *Briefings in bioinformatics*, 2020.

[38] Sudeep Pushpakom, Francesco Iorio, Patrick A Eyers, K Jane Escott, Shirley Hopper, Andrew Wells, Andrew Doig, Tim Guilliams, Joanna Latimer, Christine McNamee, et al. Drug repurposing: progress, challenges and recommendations. *Nature reviews Drug discovery*, 18(1):41–58, 2019.

[39] Salvatore Alaimo and Alfredo Pulvirenti. Network-based drug repositioning: Approaches, resources, and research directions. In *Computational Methods for Drug Repurposing*, pages 97–113. Springer, 2019.

[40] Maryam Lotfi Shahreza, Nasser Ghadiri, Sayed Rasoul Mousavi, Jaleh Varshosaz, and James R Green. A review of network-based approaches to drug repositioning. *Briefings in bioinformatics*, 19(5):878–892, 2018.

[41] Hanqing Xue, Jie Li, Haozhe Xie, and Yadong Wang. Review of drug repositioning approaches and resources. *International journal of biological sciences*, 14(10):1232, 2018.

[42] Tamer N Jarada, Jon G Rokne, and Reda Alhajj. A review of computational drug repositioning: strategies, approaches, opportunities, challenges, and directions. *Journal of Cheminformatics*, 12(1):1–23, 2020.

[43] Zheng Yao Low, Isra Ahmad Farouk, and Sunil Kumar Lal. Drug repositioning: New approaches and future prospects for life-debilitating diseases and the covid-19 pandemic outbreak. *Viruses*, 12(9):1058, 2020.

[44] Yadi Zhou, Fei Wang, Jian Tang, Ruth Nussinov, and Feixiong Cheng. Artificial intelligence in covid-19 drug repurposing. *The Lancet Digital Health*, 2020.

[45] Serguei Nabirotchkin, Alex E Peluffo, Philippe Rinaudo, Jinchao Yu, Rodolphe Hajj, and Daniel Cohen. Next-generation drug repurposing using human genetics and network biology. *Current Opinion in Pharmacology*, 2020.

[46] Patrycja Nowak-Sliwinska, Leonardo Scapozza, and Ariel Ruiz i Altaba. Drug repurposing in oncology: Compounds, pathways, phenotypes and computational approaches for colorectal cancer. *Biochimica et Biophysica Acta (BBA)-Reviews on Cancer*, 1871(2):434–454, 2019.

[47] Zhe Zhang, Li Zhou, Na Xie, Edouard C Nice, Tao Zhang, Yongping Cui, and Canhua Huang. Overcoming cancer therapeutic bottleneck by drug repurposing. *Signal transduction and targeted therapy*, 5(1):1–25, 2020.

[48] Beste Turanli, Morten Grøtli, Jan Boren, Jens Nielsen, Mathias Uhlen, Kazim Y Arga, and Adil Mardinoglu. Drug repositioning for effective prostate cancer treatment. *Frontiers in Physiology*, 9:500, 2018.

[49] RL Kruse and K Vanijcharoenkarn. Drug repurposing to treat asthma and allergic disorders: Progress and prospects. *Allergy*, 73(2):313–322, 2018.

[50] Ru-Yin Hu, Xiao-Bin Tian, Bo Li, Rui Luo, Bin Zhang, and Jin-Min Zhao. Individualized drug repositioning for rheumatoid arthritis using weighted kolmogorov–smirnov algorithm. *Pharmacogenomics and Personalized Medicine*, 12:369, 2019.

[51] Ki-Jo Kim, Navneet Rai, Minseung Kim, and Ilias Tagkopoulos. A network-based model for drug repurposing in rheumatoid arthritis. *BioRxiv*, page 335679, 2018.

[52] Gennaro Ciliberto, Rita Mancini, and Marco G Paggi. Drug repurposing against covid-19: focus on anticancer agents. *Journal of Experimental & Clinical Cancer Research*, 39:1–9, 2020.

[53] Thakur Uttam Singh, Subhashree Parida, Madhu Cholenahalli Lingaraju, Manickam Kesavan, Dinesh Kumar, and Raj Kumar Singh. Drug repurposing approach to fight covid-19. *Pharmacological Reports*, pages 1–30, 2020.

[54] Albert-Laszlo Barabasi and Zoltan N Oltvai. Network biology: understanding the cell's functional organization. *Nature reviews genetics*, 5(2):101–113, 2004.

[55] Matteo Re and Giorgio Valentini. Network-based drug ranking and repositioning with respect to drugbank therapeutic categories. *IEEE/ACM Transactions on Computational Biology and Bioinformatics*, 10(6):1359–1371, 2013.

[56] Qiaonan Duan, Corey Flynn, Mario Niepel, Marc Hafner, Jeremy L Muhlich, Nicolas F Fernandez, Andrew D Rouillard, Christopher M Tan, Edward Y Chen, Todd R Golub, et al. Lincs canvas browser: interactive web app to query, browse and interrogate lincs l1000 gene expression signatures. *Nucleic acids research*, 42(W1):W449–W460, 2014.

[57] Aravind Subramanian, Rajiv Narayan, Steven M Corsello, David D Peck, Ted E Natoli, Xiaodong Lu, Joshua Gould, John F Davis, Andrew A Tubelli, Jacob K Asiedu, et al. A next generation connectivity map: L1000 platform and the first 1,000,000 profiles. *Cell*, 171(6):1437–1452, 2017.

[58] YH Taguchi and Turki Turki. A new advanced in silico drug discovery method for novel coronavirus (sars-cov-2) with tensor decomposition-based unsupervised feature extraction. *Preprints*, 2020.

[59] Daniel Blanco-Melo, Benjamin Nilsson-Payant, Wen-Chun Liu, Rasmus Møller, Maryline Panis, David Sachs, Randy Albrecht, et al. Sars-cov-2 launches a unique transcriptional signature from in vitro, ex vivo, and in vivo systems. *BioRxiv*, 2020.

[60] Maxim V Kuleshov, Matthew R Jones, Andrew D Rouillard, Nicolas F Fernandez, Qiaonan Duan, Zichen Wang, Simon Koplev, Sherry L Jenkins, Kathleen M Jagodnik, Alexander Lachmann, et al. Enrichr: a comprehensive gene set enrichment analysis web server 2016 update. *Nucleic acids research*, 44(W1):W90–W97, 2016.

[61] Camilo Ruiz, Marinka Zitnik, and Jure Leskovec. Discovery of disease treatment mechanisms through the multiscale interactome. *bioRxiv*, 2020.

[62] Giulia Fiscon, Federica Conte, Gianpiero D'Offizi, Lorenzo Farina, and Paola Paci. Saverunner: a network-based algorithm for drug repurposing and its application to covid-19. *arXiv preprint arXiv:2006.03110*, 2020.

[63] Giulia Fiscon, Federica Conte, Lorenzo Farina, and Paola Paci. Saverunner: A network-based algorithm for drug repurposing and its application to covid-19. *PLOS Computational Biology*, 17(2):e1008686, 2021.

[64] Deisy Morselli Gysi, Ítalo Do Valle, Marinka Zitnik, Asher Ameli, Xiao Gan, Onur Varol, Helia Sanchez, Rebecca Marlene Baron, Dina Ghiassian, Joseph Loscalzo, and Albert-László Barabási. Network medicine framework for identifying drug repurposing opportunities for covid-19, 2020.

[65] David E Gordon, Gwendolyn M Jang, Mehdi Bouhaddou, Jiewei Xu, Kirsten Obernier, Kris M White, Matthew J O'Meara, Veronica V Rezelj, Jeffrey Z Guo, Danielle L Swaney, et al. A sars-cov-2 protein interaction map reveals targets for drug repurposing. *Nature*, pages 1–13, 2020.

[66] David S Wishart, Yannick D Feunang, An C Guo, Elvis J Lo, Ana Marcu, Jason R Grant, Tanvir Sajed, Daniel Johnson, Carin Li, Zinat Sayeeda, et al. Drugbank 5.0: a major update to the drugbank database for 2018. *Nucleic acids research*, 46(D1):D1074–D1082, 2018.

[67] Yadi Zhou, Yuan Hou, Jiayu Shen, Yin Huang, William Martin, and Feixiong Cheng. Network-based drug repurposing for novel coronavirus 2019-ncov/sars-cov-2. *Cell discovery*, 6(1):1–18, 2020.

[68] Sepideh Sadegh, Julian Matschinske, David B. Blumenthal, Gihanna Galindez, Tim Kacprowski, Markus List, Reza Nasirigerdeh, Mhaned Oubounyt, Andreas Pichlmair, Tim Daniel Rose, and et al. Exploring the sars-cov-2 virus-host-drug interactome for drug repurposing. *Nature Communications*, 11(1), Jul 2020.

[69] Nicolas Alcaraz, Hande Kücük, Jochen Weile, Anil Wipat, and Jan Baumbach. Keypathwayminer: detecting case-specific biological pathways using expression data. *Internet Mathematics*, 7(4):299–313, 2011.

[70] Raghvendra Mall, Abdurrahman Elbasir, Hossam Al Meer, Sanjay Chawla, and Ehsan Ullah. Data-driven drug repurposing for covid-19. *ChemRxiv chemrxiv*, 12661103:v1, 2020.

[71] P Chellapandi and S Saranya. Genomics insights of sars-cov-2 (covid-19) into target-based drug discovery. *Medicinal Chemistry Research*, pages 1–15, 2020.

[72] Sangjae Seo, Jung Woo Park, Dosik An, Junwon Yoon, Hyojung Paik, and Soonwook Hwang. Supercomputer-aided Drug Repositioning at Scale: Virtual Screening for SARS-CoV-2 Protease Inhibitor. *ChemrXiv*, 4 2020.

[73] Aleix Gimeno, Júlia Mestres-Truyol, María José Ojeda-Montes, Guillem Macip, Bryan Saldivar-Espinoza, Adrià Cereto-Massagué, Gerard Pujadas, and Santiago Garcia-Vallvé. Prediction of novel inhibitors of the main protease (m-pro) of sars-cov-2 through consensus docking and drug reposition. *International Journal of Molecular Sciences*, 21(11):3793, 2020.

[74] Bhumi Shah, Palmi Modi, and Sneha R Sagar. In silico studies on therapeutic agents for covid-19: Drug repurposing approach. *Life Sciences*, page 117652, 2020.

[75] Ammar D Elmezayen, Anas Al-Obaidi, Alp Tegin Şahin, and Kemal Yelekçi. Drug repurposing for coronavirus (covid-19): in silico screening of known drugs against coronavirus 3cl hydrolase and protease enzymes. *Journal of Biomolecular Structure and Dynamics*, pages 1–13, 2020.

[76] Junmei Wang. Fast identification of possible drug treatment of coronavirus disease-19 (covid-19) through computational drug repurposing study. *Journal of Chemical Information and Modeling*, 2020.

[77] Alfonso Trezza, Daniele Iovinelli, Annalisa Santucci, Filippo Prischi, and Ottavia Spiga. An integrated drug repurposing strategy for the rapid identification of potential sars-cov-2 viral inhibitors. *Scientific reports*, 10(1):1–8, 2020.

[78] Sovesh Mahapatra, Prathul Nath, Manisha Chatterjee, Neeladrisingha Das, Deepjyoti Kalita, Partha Roy, and Soumitra Satapathi. Repurposing therapeutics for covid-19: Rapid prediction of commercially available drugs through machine learning and docking. *medRxiv*, 2020.

[79] Laura Riva, Shuofeng Yuan, Xin Yin, Laura Martin-Sancho, Naoko Matsunaga, Sebastian Burgstaller, Lars Pache, Paul De Jesus, Mitchell V Hull, Max Chang, et al. A large-scale drug repositioning survey for sars-cov-2 antivirals. *bioRxiv*, 2020.

[80] Laura Riva, Shuofeng Yuan, Xin Yin, Laura Martin-Sancho, Naoko Matsunaga, Lars Pache, Sebastian Burgstaller-Muehlbacher, Paul D De Jesus, Peter Teriete, Mitchell V Hull, et al. Discovery of sars-cov-2 antiviral drugs through large-scale compound repurposing. *Nature*, 586(7827):113–119, 2020.

[81] Carmen Mirabelli, Jesse W Wotring, Charles J Zhang, Sean M McCarty, Reid Fursmidt, Namrata S Kadambi, Anya T Amin, Teresa R O'Meara, Carla D Pretto-Kernahan, Jason R Spence, et al. Morphological cell profiling of sars-cov-2 infection identifies drug repurposing candidates for covid-19. *bioRxiv*, 2020.

[82] Beatriz Luna, Marcelino Ramírez, and Edgardo Galán. Network analysis and disease subnets for the sars-cov-2/human interactome, 2020.

[83] Xiangxiang Zeng, Xiang Song, Tengfei Ma, Xiaoqin Pan, Yadi Zhou, Yuan Hou, Zheng Zhang, Kenli Li, George Karypis, and Feixiong Cheng. Repurpose open data to discover therapeutics for covid-19 using deep learning. *Journal of proteome research*, 19(11):4624–4636, 2020.

[84] Paola Stolfi, Luigi Manni, Marzia Soligo, Davide Vergni, and Paolo Tieri. Designing a network proximity-based drug repurposing strategy for covid-19. *Frontiers in cell and developmental biology*, 8, 2020.

[85] Claudio Cavasotto and Juan Di Filippo. In silico drug repurposing for covid-19: Targeting sars-cov-2 proteins through docking and quantum mechanical scoring. *ChemRxiv*, 2020.

[86] Huiyuan Chen, Feixiong Cheng, and Jing Li. idrug: Integration of drug repositioning and drug-target prediction via cross-network embedding. *PLoS computational biology*, 16(7):e1008040, 2020.

[87] Jie Huang, Jiazhou Chen, Bin Zhang, Lei Zhu, and Hongmin Cai. Evaluation of gene–drug common module identification methods using pharmacogenomics data. *Briefings in Bioinformatics*, 2020.

[88] Carlos Loucera, Marina Esteban-Medina, Kinza Rian, Matías M Falco, Joaquín Dopazo, and María Peña-Chilet. Drug repurposing for covid-19 using machine learning and mechanistic models of signal transduction circuits related to sars-cov-2 infection. *Signal transduction and targeted therapy*, 5(1):1–3, 2020.

[89] Yonghyun Nam, Jae-Seung Yun, Seung Mi Lee, Ji Won Park, Ziqi Chen, Brian Lee, Anurag Verma, Xia Ning, Li Shen, and Dokyoon Kim. Network reinforcement driven drug repurposing for covid-19 by exploiting disease-gene-drug associations. *arXiv preprint arXiv:2008.05377*, 2020.

[90] Yiyue Ge, Tingzhong Tian, Sulin Huang, Fangping Wan, Jingxin Li, Shuya Li, Hui Yang, Lixiang Hong, Nian Wu, Enming Yuan, et al. A data-driven drug repositioning framework discovered a potential therapeutic agent targeting covid-19. *BioRxiv*, 2020.

[91] Francesco Iorio, Roshan L Shrestha, Nicolas Levin, Viviane Boilot, Mathew J Garnett, Julio Saez-Rodriguez, and Viji M Draviam. A semi-supervised approach for refining transcriptional signatures of drug response and repositioning predictions. *PloS one*, 10(10):e0139446, 2015.

[92] Mike Fang, Brian Richardson, Cheryl M Cameron, Jean-Eudes Dazard, and Mark J Cameron. Drug perturbation gene set enrichment analysis (dpgsea): a new transcriptomic drug screening approach. *BMC bioinformatics*, 22(1):1–14, 2021.

[93] M Tomazou, M M Bourdakou, G Minadakis, M Zachariou, A Oulas, E Karatzas, E Loizidou, A Kakouri, C Christodoulou, K Savva, M Zanti, A Onisiforou, S Afxenti, J Richter, C G Christodoulou, T Kyprianou, G Kolios, N Dietis, and G M Spyrou. Multi-omics data integration and network-based analysis drives a multiplex drug repurposing approach to a shortlist of candidate drugs against COVID-19. *Briefings in Bioinformatics*, 05 2021. bbab114.

[94] Beril Tutuncuoglu, Merve Cakir, Jyoti Batra, Mehdi Bouhaddou, Manon Eckhardt, David E Gordon, and Nevan J Krogan. The landscape of human cancer proteins targeted by sars-cov-2. *Cancer discovery*, 10(7):916–921, 2020.

[95] Eugene Muratov and Alexey Zakharov. Viribus unitis: Drug combinations as a treatment against covid-19. *ChemRxiv*, 2020.

[96] Laura Artigas, Mireia Coma, Pedro Matos-Filipe, Joaquim Aguirre-Plans, Judith Farrés, Raquel Valls, Narcis Fernandez-Fuentes, Juan de la Haba-Rodriguez, Alex Olvera, Jose Barbera, et al. In-silico drug repurposing study predicts the combination of pirfenidone and melatonin as a promising candidate therapy to reduce sars-cov-2 infection progression and respiratory distress caused by cytokine storm. *PloS one*, 15(10):e0240149, 2020.

[97] Tesia Bobrowski, Lu Chen, Richard T Eastman, Zina Itkin, Paul Shinn, Catherine Z Chen, Hui Guo, Wei Zheng, Sam Michael, Anton Simeonov, et al. Discovery of synergistic and antagonistic drug combinations against sars-cov-2 in vitro. *biorXiv*, 2020. doi:10.1101/2020.06.29.178889.

[98] Tesia Bobrowski, Lu Chen, Richard T Eastman, Zina Itkin, Paul Shinn, Catherine Z Chen, Hui Guo, Wei Zheng, Sam Michael, Anton Simeonov, et al. Synergistic and antagonistic drug combinations against sars-cov-2. *Molecular Therapy*, 29(2):873–885, 2021.

[99] Wengong Jin, Regina Barzilay, and Tommi Jaakkola. Modeling drug combinations based on molecular structures and biological targets. *arXiv preprint arXiv:2011.04651*, 2020.

[100] Feixiong Cheng, István A Kovács, and Albert-László Barabási. Network-based prediction of drug combinations. *Nature communications*, 10(1):1–11, 2019.

[101] Marinka Zitnik, Monica Agrawal, and Jure Leskovec. Modeling polypharmacy side effects with graph convolutional networks. *Bioinformatics*, 34(13):i457–i466, 2018.

[102] Aled Edwards. What are the odds of finding a covid-19 drug from a lab repurposing screen? *Journal of Chemical Information and Modeling*, 2020.

[103] Adam S Brown and Chirag J Patel. A review of validation strategies for computational drug repositioning. *Briefings in bioinformatics*, 19(1):174–177, 2018.
